# Genetic and epigenetic alterations in oral potentially malignant disorders: A cross-sectional clinical study

**DOI:** 10.6026/973206300213051

**Published:** 2025-09-30

**Authors:** Akanksha Baheti, Timbadiya Vijaykumar Mansukhbhai, Parth M. Raviya, Kajal Shilu, Jyoti Singh, Yadavalli Guruprasad

**Affiliations:** 1 Department of Esthetics and Restorative Dentistry, Associate Dentist, Dental Experts Clinic, Chicago, IL USA; 2 Department of Oral Pathology and Microbiology, Rishiraj College of Dental Sciences and Research Centre, Bhopal, Madhya Pradesh, India; 3 Department of Oral & Maxillofacial Surgery, Government Dental College and Hospital, Jamnagar, Gujarat, India; 4 Department of Oral Medicine & Radiology, Government Dental College and Hospital, Jamnagar, Gujarat, India; 5 Department of Public Health Dentistry, Santosh Dental College, Santosh Deemed to be University, Delhi NCR, India; 6 Department of Oral & Maxillofacial Surgery, Government Dental College and Research Institute, VIMS Campus, Cantonment, Ballari, Karnataka, India

**Keywords:** Oral potentially malignant disorders, genetic alteration, epigenetic modification, TP53, DNA methylation, p16INK4a, oral cancer risk

## Abstract

The prevalence and spectrum of genetic and epigenetic alterations in oral potentially malignant disorders (OPMDs) is of interest. Hence, a total of 132 patients with clinically and histopathologically diagnosed OPMDs were evaluated for key molecular changes, including TP53 mutations, promoter methylation of tumor suppressor genes and global DNA hypomethylation. Salivary and tissue samples were analyzed using PCR, methylation-specific PCR (MSP) and immunohistochemistry. TP53 mutations and p16INK4a promoter hypermethylation were significantly associated with severe dysplasia and higher malignant transformation risk. Thus, integrating molecular profiling into OPMD evaluation could improve risk stratification and early intervention.

## Background:

Oral potentially malignant disorders (OPMDs) encompass a group of mucosal lesions with an increased risk of progressing to oral squamous cell carcinoma (OSCC), the most common malignancy of the oral cavity [1]. Oral squamous cell carcinoma (OSCC) is a prevalent and significant type of oral cancer that has far-reaching health implications worldwide [2]. Despite advances in clinical diagnosis and histopathological grading, predicting which lesions will undergo malignant transformation remains a major challenge [3]. Traditional assessments based on clinical appearance and epithelial dysplasia often fails to capture the underlying biological behavior of the lesion [4]. Recent research has highlighted the importance of molecular alterations-both genetic and epigenetic-in the initiation and progression of OPMDs [5]. Genetic mutations such as those in the TP53 tumor suppressor gene can lead to unchecked cellular proliferation and resistance to apoptosis [6]. Similarly, epigenetic changes like promoter hypermethylation of genes such as p16INK4a and MGMT, as well as global hypomethylation, contribute to gene silencing and genomic instability, respectively [7]. These molecular events often precede morphological changes and may serve as early indicators of malignant potential [8]. While numerous studies have evaluated individual biomarkers, comprehensive clinical studies assessing a panel of genetic and epigenetic changes in OPMDs remain limited [9]. Therefore, it is of interest to evaluate the prevalence of selected molecular alterations in patients with histologically confirmed OPMDs and correlate them with dysplasia severity.

## Materials and Methods:

This cross-sectional observational study was conducted over a period of 18 months in the Department of Oral Medicine and Pathology at a tertiary care center. A total of 132 patients aged 18 years and above, presenting with clinically suspected oral potentially malignant disorders (OPMDs) such as leukoplakia, erythroplakia, oral submucous fibrosis (OSMF) and lichen planus, were enrolled following written informed consent. Diagnosis of OPMDs was confirmed through incisional biopsy and histopathological evaluation, with grading of dysplasia done according to the WHO 2021 classification. Patients with prior history of oral cancer, chemotherapy, radiotherapy, or systemic conditions that could influence DNA methylation status (*e.g.*, autoimmune diseases, chronic infections) were excluded from the study. Demographic details, tobacco usage, lesion type and site were recorded. Tissue samples obtained during biopsy were preserved for molecular analysis and unstimulated saliva samples were also collected in sterile containers. Genomic DNA was extracted from both tissue and saliva using the Qiagen DNA extraction kit. Mutation analysis for the TP53 gene (exons 5-8) was performed using PCR followed by Sanger sequencing. Epigenetic analysis included promoter methylation assessment of p16INK4a and MGMT using methylation-specific PCR (MSP) and global DNA methylation levels were quantified using ELISA-based 5-methylcytosine quantification. Immunohistochemical expression of p53 and p16 proteins was also evaluated in tissue sections. Statistical analysis was performed using SPSS version 26.0. Associations between molecular alterations and clinicopathological features (type of OPMD, dysplasia grade, tobacco use) were evaluated using Chi-square or Fisher's exact test, with p-values <0.05 considered statistically significant. Multivariate logistic regression was applied to assess predictors of high-risk lesions.

## Results:

A total of 132 patients with clinically and histologically confirmed OPMDs were included in the study. The mean age was 46.2 ± 12.8 years, with a male predominance (M:F = 1.6:1). Leukoplakia was the most common lesion type, followed by oral submucous fibrosis (OSMF) and erythroplakia. Molecular analysis revealed significant frequencies of TP53 mutations and promoter hypermethylation of tumor suppressor genes, particularly in lesions with moderate to severe dysplasia. Table 1 (see PDF) shows that most patients were in the 41-60 age groups and tobacco use was present in over 70% of cases. Table 2 (see PDF) shows Leukoplakia and OSMF were the most prevalent OPMDs in the study population. Table 3 (see PDF) shows moderate and severe dysplasia were more frequently associated with leukoplakia and erythroplakia. Table 4 (see PDF) shows TP53 gene mutations were detected in nearly half of the moderate-to-severe dysplasia cases. Table 5 (see PDF) shows promoter hypermethylation of p16INK4a was most frequent in severe dysplasia. Table 6 (see PDF) shows MGMT promoter hypermethylation was less frequent but associated with higher-grade lesions. Table 7 (see PDF) shows global DNA hypomethylation increased with the severity of dysplasia. Table 8 (see PDF) shows p53 protein overexpression correlated with the presence of TP53 mutations. Table 9 (see PDF) shows patients with tobacco use showed a significantly higher frequency of genetic and epigenetic alterations. Table 10 (see PDF) shows multiple molecular alterations were found to co-occur more often in high-risk lesions.

## Discussion:

This study provides comprehensive insight into the genetic and epigenetic landscape of oral potentially malignant disorders (OPMDs) and their correlation with dysplasia severity. Our findings highlight a significant association between TP53 mutations and increasing grades of epithelial dysplasia, with mutation prevalence rising from 19.4% in mild to 80% in severe dysplasia. This supports the critical role of TP53 as a tumor suppressor gene whose dysfunction is a hallmark of malignant progression in oral mucosa. The overexpression of p53 protein detected by immunohistochemistry corresponded well with TP53 mutations, reaffirming its diagnostic utility [10]. Promoter hypermethylation of p16INK4a and MGMT was markedly higher in moderate and severe dysplasia, reflecting the silencing of these tumor suppressor genes during carcinogenesis. The high methylation rate of p16INK4a (83.3% in severe dysplasia) aligns with previous studies indicating that epigenetic silencing of cell cycle regulators is a key event in oral carcinogenesis. Global DNA hypomethylation, which contributes to chromosomal instability, also increased significantly with dysplasia grade, confirming its role as an early epigenetic alteration facilitating malignant transformation [11]. It is precisely important to risk-stratify the patients based on numerous factors, like the size, clinical appearance, and histology of the lesion to provide appropriate counseling and screening for higher-risk individuals. Further, patients with a habit of tobacco chewing, areca nut consumption, alcoholism, smoking, and drug abuse are at high risk [12]. The predominance of tobacco users among cases with molecular alterations emphasizes the carcinogenic influence of tobacco on genetic and epigenetic damage. Tobacco's ability to induce DNA mutations and alter methylation patterns likely accelerates the risk of progression in OPMDs. The co-occurrence of multiple alterations in high-grade lesions suggests a cumulative effect of genetic and epigenetic changes driving malignant potential [13]. Overall, this study underscores the limitations of relying solely on clinical and histopathological evaluation for risk assessment. Molecular profiling of OPMDs provides critical prognostic information that can guide early intervention and surveillance strategies. Incorporation of TP53 mutation analysis and methylation markers into routine diagnostic workflows may improve prediction of malignant transformation and enable personalized patient management [14]. Integrating genetic and epigenetic markers with traditional diagnostics offers a promising approach for improved risk stratification in OPMDs, potentially reducing the burden of oral cancer through earlier detection and tailored treatment.

## Conclusion:

Genetic mutations, particularly in TP53, along with epigenetic alterations like promoter hypermethylation of p16INK4a and MGMT, are strongly associated with the severity of dysplasia in oral potentially malignant disorders. These molecular changes can serve as valuable biomarkers for identifying high-risk lesions with greater malignant transformation potential. Incorporating molecular profiling into routine evaluation of OPMDs may enhance early detection and improve patient management strategies.
